# Issues and Limitations on the Road to Fair and Inclusive AI Solutions for Biomedical Challenges

**DOI:** 10.3390/s25010205

**Published:** 2025-01-02

**Authors:** Oliver Faust, Massimo Salvi, Prabal Datta Barua, Subrata Chakraborty, Filippo Molinari, U. Rajendra Acharya

**Affiliations:** 1 School of Computing and Information Science, Anglia Ruskin University, Cambridge Campus, Cambridge CB1 1PT, UK; 2 PoliToBIOMed Lab, Biolab, Department of Electronics and Telecommunications, Politecnico di Torino, Corso Duca Degli Abruzzi 24, 10129 Turin, Italy; massimo.salvi@polito.it (M.S.); filippo.molinari@polito.it (F.M.); 3 Cogninet Australia, Sydney, NSW 2010, Australia; prabal.barua@unisq.edu.au; 4 School of Business (Information Systems), University of Southern Queensland, Toowoomba, QLD 4350, Australia; 5 Faculty of Engineering and Information Technology, University of Technology Sydney, Sydney, NSW 2007, Australia; 6 Australian International Institute of Higher Education, Sydney, NSW 2000, Australia; 7 School of Science and Technology, University of New England, Armidale, NSW 2351, Australia; subrata.chakraborty@une.edu.au; 8 School of Biosciences, Taylor’s University, Subang Jaya 47500, Malaysia; 9 School of Computing, SRM Institute of Science and Technology, Kattankulathur 603203, India; 10 School of Science and Technology, Kumamoto University, Kumamoto 860-8555, Japan; 11 Sydney School of Education and Social Work, University of Sydney, Camperdown, NSW 2050, Australia; 12 Centre for Advanced Modelling and Geospatial Information Systems (CAMGIS), Faculty of Engineering and Information Technology, University of Technology Sydney, Ultimo, NSW 2007, Australia; 13 Griffith Business School, Griffith University, Brisbane, QLD 4111, Australia; 14 School of Mathematics, Physics and Computing, University of Southern Queensland, Springfield, QLD 4300, Australia; rajendra.acharya@unisq.edu.au; 15 Centre for Health Research, University of Southern Queensland, Ipswich, QLD 4305, Australia

**Keywords:** bias, noise, inclusive AI, trust, explainability, system design

## Abstract

Objective: In this paper, we explore the correlation between performance reporting and the development of inclusive AI solutions for biomedical problems. Our study examines the critical aspects of bias and noise in the context of medical decision support, aiming to provide actionable solutions. Contributions: A key contribution of our work is the recognition that measurement processes introduce noise and bias arising from human data interpretation and selection. We introduce the concept of “noise-bias cascade” to explain their interconnected nature. While current AI models handle noise well, bias remains a significant obstacle in achieving practical performance in these models. Our analysis spans the entire AI development lifecycle, from data collection to model deployment. Recommendations: To effectively mitigate bias, we assert the need to implement additional measures such as rigorous study design; appropriate statistical analysis; transparent reporting; and diverse research representation. Furthermore, we strongly recommend the integration of uncertainty measures during model deployment to ensure the utmost fairness and inclusivity. These comprehensive recommendations aim to minimize both bias and noise, thereby improving the performance of future medical decision support systems.

## 1. Introduction

While artificial intelligence (AI) continues to make remarkable advances in medical decision support, we argue that current approaches to ensure fairness and inclusivity fall short when addressing the unique challenges of the medical domain. This position paper argues that a fundamental rethinking of how we approach fairness in medical AI is necessary due to the high stakes involved in medical judgements, the complexity of multi-modal medical data, and the deeply embedded biases in current healthcare systems.

Achieving fairness in medical AI not only requires addressing biases but also confronting the inherent complexities of real-world medical data. Engineers and computer scientists strive to maintain control over the operating environment for their systems and software programs. In an ideal scenario, these solutions exhibit perfect repeatability, with no variations between operators or conditions. However, practical problem-solving inevitably widens the gap between a system’s performance in controlled laboratory settings and its real-world deployment performance [1]. This performance gap is particularly significant in the context of medical decision support systems [2,3,4]. The extent of the performance gap is directly linked to the level of control over the data acquisition process in the medical environment [5]. Unfortunately, the absence of objective measures hampers the assessment of the quality of medical data acquisition and control [6,7]. The presence of unavoidable noise during measurements introduces uncertainty. In healthcare applications, noise can manifest as variations in disease symptoms, which subsequently affect the measurement data collected from patients at specific points in time.

AI models aim to emulate human decision-making processes in providing medical decision support [8]. This approach is philosophically justified by analogies between human thinking and machine decision-making. Medical professionals navigate uncertain environments with limited control, their expertise serving as invaluable knowledge sources [9,10]. Capturing this knowledge involves human experts labeling data, inevitably introducing subjectivity and bias, which limits the transferability of results from medical decision support systems [11,12]. Data and their interpretation stem from individual or group decisions, influenced by intentions about what to measure, when to measure, and how to interpret [13,14].

Bias, a systematic deviation from objectivity and fairness, can arise from various sources in medical measurements, including selection bias [15,16], measurement bias [17,18], confounding bias [19], and cultural or demographic bias [20]. AI systems can amplify these biases if trained on biased datasets or if algorithms have inherent biases [21]. This can perpetuate existing biases, introduce new ones, and lead to unfair outcomes in diagnostic pathways. The challenge for engineers is developing medical decision support systems that effectively replicate professional decision-making while mitigating biases. This requires effective methods for assessing decision support quality [22,23], with algorithm design and quality measures being fair, inclusive, and comprehensive [24].

In this paper, we adopt the position that quality design and reporting for AI solutions is a multifaceted undertaking. It is necessary to incorporate mitigation measures against bias and noise during AI development and include uncertainty measures during model deployment to ensure fairness and inclusivity. These measures should be used to build trust in the model so that there is a good level of confidence before the clinical validation.

The structure of this document is as follows. The next section outlines the methods used for developing medical decision support systems, covering the basics of AI, with a specific emphasis on state-of-the-art quality measures. Finally, we discuss the implications of our position and provide recommendations for future research and development efforts.

## 2. Methods

In this section, we provide a critical analysis of various medical decision support technologies, and the methods used to assess their performance. While these technologies and methods are well established, we argue that their application in medical contexts presents unique challenges that are often overlooked in standard performance assessments.

**Figure 1:**
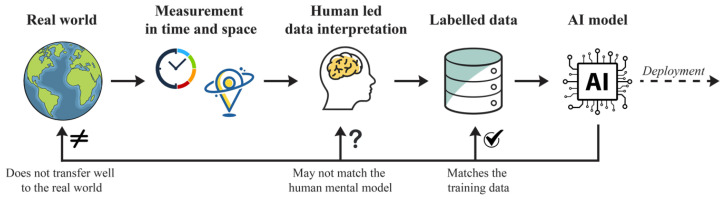
Block diagram illustrating the components and steps involved in the development of a medical decision support system. The diagram highlights four interconnected stages: measurement (introducing system noise), human-led data interpretation (incorporating expert knowledge and potential biases), AI model creation (development and validation), and model deployment (clinical implementation). The connections between components emphasize how noise and bias propagate throughout the system, influencing both the development process and final performance.

Figure 1 presents an abstract block diagram of a medical decision support system, encompassing crucial elements such as measurement, human-led data interpretation, AI model creation, and model deployment. This holistic view allows us to examine how noise and bias propagate through the entire system, affecting overall performance and fairness.

We pay particular attention to the measurement block, which introduces noise that impacts AI model performance [23], and the human data interpretation block, which introduces bias into decision support systems. These elements are often treated as separate issues in traditional AI development, but they are intrinsically linked in medical contexts and must be addressed simultaneously. While we discuss AI model creation and associated performance measures [24], our analysis goes beyond standard metrics. We critically examine how these measures may fail to capture the full complexity of medical decision-making and propose alternative approaches that better account for the unique challenges in healthcare settings.

A unique aspect of our analysis is the recognition of the cyclical relationship between measurement noise and interpretation bias in medical contexts. While traditional approaches treat these as independent challenges, we propose that they form a feedback loop: measurement noise influences human interpretation strategies, leading to compensatory biases, while biased interpretation affects future measurement protocols, introducing systematic noise. This interdependence creates what we term a “noise-bias cascade” that conventional AI evaluation frameworks fail to address. This perspective shifts the focus from treating noise and bias as separate technical challenges to understanding them as interlinked components of the medical decision-making ecosystem.

### 2.1. Measurement

Medical data measurement is a fundamental process that involves systematically and quantitatively assessing various aspects of health, diseases, treatments, or other relevant variables within the field of medicine. It plays a crucial role in evidence-based medicine, clinical research, healthcare quality improvement, and personalized patient care. The accurate and reliable collection, recording, and analysis of data using specific measurement tools, techniques, or instruments are vital for ensuring the integrity and usefulness of medical information.

The diversity of medical data types (ranging from physiological measurements and clinical observations to patient-reported outcomes, imaging data, and genetic information [25,26,27]) creates a multifaceted landscape that AI models must navigate. This complexity exacerbates the impact of noise and bias in ways that are distinct from other domains of AI application. Bias in medical data collection often stems from unrepresentative or exclusionary practices. For example, wearable devices calibrated primarily for lighter skin tones may produce inaccurate readings for individuals with darker skin, introducing systematic bias in the data [28]. Similarly, geographic disparities lead to uneven data representation, as rural areas often lack diagnostic infrastructure, resulting in lower-quality or incomplete datasets. To address these challenges, targeted data collection strategies, such as oversampling underrepresented groups and standardizing measurement protocols, are essential. Federated learning can help mitigate biases by training AI models across decentralized datasets without compromising patient privacy. For example, a federated model, trained on patient records from hospitals across diverse regions, can account for demographic and geographic variations, reducing biases in AI predictions. Privacy-preserving techniques, such as differential privacy and secure multiparty computation, further enhance this approach by safeguarding sensitive information during the training process. These advancements ensure that AI models remain inclusive while maintaining compliance with ethical and legal standards.

While strategies such as sensor fusion and uncertainty quantification [29,30] can improve AI model robustness against noise, we argue that these approaches must be tailored specifically for medical applications. Standard validation techniques for measurement tools and data collection procedures [31,32,33] may not fully capture the nuanced ways in which bias can manifest in medical contexts.

There is a need for a more comprehensive approach to ensuring the accuracy, reliability, and validity of medical data measurement for AI applications. Upholding rigorous standards and practices in medical data measurement is not just about improving data quality, but also about ensuring equitable and safe healthcare outcomes for all populations [34].

### 2.2. Human-Led Data Interpretation

The application of clinical knowledge and expertise is a crucial aspect of human-based medical data interpretation. By drawing upon their clinical experience and understanding of medical concepts, pathophysiology, and evidence-based practices, medical experts provide valuable insights that complement automated approaches [35,36]. We argue that the integration of this human expertise with AI systems in healthcare settings introduces complexities that go beyond those seen in other domains.

While human expertise is invaluable, it also introduces bias due to subjective judgments and cognitive limitations. For example, clinicians may consciously or unconsciously interpret diagnostic data differently based on a patient’s demographic characteristics (age, gender, ethnicity), potentially influencing subsequent AI predictions. This interpretation bias can skew the performance of AI models if these subjective decisions are incorporated into training datasets. Bias quantification methods, such as comparing model performance across subgroups (e.g., by demographic), can help detect these biases. Statistical tests or fairness metrics (such as group-specific precision, recall, or AUC) can be employed to measure how well the model performs for different populations, highlighting potential biases introduced by human-led interpretation.

Another significant concern arises from the labeling of training data. Human error or inconsistency during data labeling, especially in large-scale datasets, can create label noise, further diminishing the model’s capacity to generalize. Therefore, it is recommended to apply robustness measures in AI models that account for noisy labels, such as active learning or semi-supervised learning approaches.

### 2.3. Model Creation

Model creation is a multi-step process that involves several key stages: preprocessing, training, and testing. The first step in creating a model is preprocessing the data to prepare them for analysis by filtering, cleaning, and transforming them into a suitable format for the model. Next, the model is trained on the preprocessed data, with its parameters adjusted iteratively to optimize its performance. Once the model has been trained, it is evaluated on a separate set of data to test its accuracy and generalizability. This testing phase is essential for ensuring that the model is not overfitting to the training data. Performance measures play a crucial role in providing feedback to guide the model creation process [1,37].

#### 2.3.1. Preprocessing

During preprocessing, careful attention must be paid to addressing the potential for bias arising from unrepresentative or incomplete data. It is important to employ techniques that can detect and mitigate biases in AI models [38]. One approach involves utilizing fairness metrics and evaluation methods to identify any disparate impacts that may exist within the system. Once biases are identified, modifications can be made to the algorithms to reduce or eliminate the bias. This can involve adjusting the training data, developing algorithms that are more sensitive to fairness considerations, or implementing post-processing techniques to ensure fairness in decision-making [39]. Furthermore, it is crucial to involve diverse and representative stakeholders throughout the development and evaluation process. This includes considering the perspectives and expertise of individuals from different demographic groups to ensure that potential biases are identified and addressed comprehensively [40].

#### 2.3.2. Training and Testing

At an abstract level, AI algorithms in the field of healthcare are typically based on either supervised or unsupervised learning approaches [41]. Unsupervised learning methods discover hidden data structures and patterns within medical datasets [42], providing insights into disease hotspots, interactions between multiple diseases in multimorbid patients, and the socioeconomic implications. This exploration through unsupervised learning enables knowledge generation, which has the potential to drive medical progress. However, the interpretation of these discovered patterns requires careful consideration, based on medical knowledge, to avoid spurious correlations or clinically irrelevant findings.

Supervised learning aims to tap into this existing knowledge by training and testing models using labeled data [43]. These labels result from human-led data interpretation, as discussed in Section 2.2. On a technical level, the first step in conducting supervised learning is to divide the available data into training and test sets. The training set is utilized by the algorithm to learn and build a model, while the test set is employed to evaluate the model’s performance. By splitting the data in this manner, we can assess how well the trained model generalizes to new, unseen data. However, this process tends to preserve and sometimes exacerbate existing biases. For example, training on imbalanced datasets without bias correction leads to models that systematically underperform for minority groups. Similarly, validation bias—when test sets fail to represent the full diversity of the population—can result in misleading performance metrics that may not reflect real-world clinical performance. Recent advancements, such as federated learning [44] and privacy-preserving AI [45], allow the training of models across decentralized datasets while preserving privacy. These approaches mitigate demographic biases and increase inclusivity by incorporating data from diverse, distributed sources.

To ensure fair and inclusive model development, we advocate for the pervasive use of fairness metrics during both training and testing. Techniques such as group-specific accuracy, equalized odds, or demographic parity should be incorporated into the evaluation process to identify disparities in model outcomes across subgroups. Additionally, the use of cross-validation with demographically stratified data can ensure that the model is tested on a broad and inclusive sample. This might result in a more accurate reflection of its performance in diverse real-world applications.

#### 2.3.3. Performance Measures

Performance measures play a critical role in assessing the effectiveness and efficiency of medical AI. These measures provide quantitative evaluations of how well an AI algorithm performs its intended task, enabling comparisons between different algorithms or variations of the same algorithm [46,47]. The following list introduces commonly used AI performance measures for medical applications:

1. Accuracy is a widely employed performance measure, particularly in classification tasks. It calculates the percentage of correctly classified instances out of the total number of instances. While accuracy is essential, it may not offer a comprehensive view of the algorithm’s performance, especially when dealing with imbalanced datasets.
2. Precision and recall are often utilized in binary classification problems. Precision measures the proportion of correctly predicted positive instances out of all instances predicted as positive, while recall measures the proportion of correctly predicted positive instances out of all actual positive instances [48]. Precision and recall are commonly combined into a single measure called the F1 score, which provides a balanced evaluation of both precision and recall.
3. Area under the curve (AUC) is commonly employed in binary classification problems to assess the performance of a classifier’s receiver operating characteristic (ROC) curve. It quantifies the classifier’s ability to rank positive instances higher than negative instances across different classification thresholds [49]. AUC values range from 0.5 (random guessing) to 1.0 (perfect classification).
4. Mean absolute error (MAE) and root mean squared error (RMSE): These measures are frequently used in regression tasks to evaluate the prediction accuracy of continuous variables. MAE calculates the average absolute difference between predicted and actual values, while RMSE calculates the square root of the average squared difference [50]. Lower values indicate better performance.
5. Computational efficiency: In addition to accuracy measures, assessing computational efficiency is crucial. It evaluates the algorithm’s speed and usage of resources, such as memory and processing power [51]. Performance measures like training time, prediction time, and memory consumption can be employed to evaluate the efficiency of AI algorithms.

These standard performance measures require careful interpretation based on domain-specific factors. For instance, in many medical applications, false negatives (missed diagnoses) may have more severe consequences than false positives, making recall potentially more critical than precision in certain contexts. The choice of performance measures depends on the specific medical problem, characteristics of the dataset, and desired outcomes. We recommend using a combination of performance measures to establish a comprehensive evaluation of an AI algorithm’s performance, particularly in medical applications where multiple aspects of performance may be crucial [52].

In the context of inclusive medical AI, it is necessary to extend performance measures to account for fairness and bias. Metrics such as equalized opportunity and disparity impact can measure model performance across different demographic groups, ensuring that the decision support model does not disproportionately benefit or harm any group. Shapley values and counterfactual fairness tests are useful in assessing whether certain input factors (e.g., race or gender) unduly influence model predictions, providing a mechanism for bias detection and mitigation.

When evaluating the model performance based on noisy data, traditional error metrics such as mean absolute error (MAE) or root mean squared error (RMSE) should be complemented with uncertainty quantification and noise-tolerant measures. Continuous evaluation and refinement of these performance measures is necessary to ensure accurate and meaningful assessments of AI algorithms, considering both potential biases and real-world impact on patient outcomes.

### 2.4. Deployment

Medical decision support systems are created by deploying a trained AI model into a production environment within a healthcare system. This process involves making the AI model operational and accessible to healthcare professionals for use in diagnosing diseases, predicting outcomes, assisting in treatment decisions, or other relevant applications. By providing a second diagnostic opinion or automating screening processes, these systems have the potential to free up resources and make decision processes more coherent. The deployment of an AI model in the medical domain typically follows these steps:

1. Model selection: The performance measures, discussed in Section 2.3.3, provide an objective basis for selecting a model for deployment.
2. Infrastructure setup: The necessary infrastructure is established to support the deployment of the AI model in the medical setting. This includes ensuring compliance with privacy regulations [53], implementing data security measures, and addressing any specific requirements for handling sensitive patient information [54].
3. Integration with healthcare systems: The AI model is integrated into existing healthcare systems, such as electronic health record (EHR) systems, medical imaging platforms, or clinical decision support tools. This integration enables seamless data exchange and interaction between the AI model and healthcare professionals [55].
4. Data access and pre-processing: The AI model is connected to relevant data sources, such as patient records, medical imaging archives, or real-time monitoring devices. Data preprocessing steps may be implemented to standardize, clean, and anonymize the data while preserving their integrity and privacy [56,57,58].
5. Testing and validation in the real-world setting: The deployed AI model undergoes extensive testing and validation in real-world medical scenarios. Its performance, accuracy, and safety are evaluated, and necessary adjustments are made to ensure optimal performance and patient safety.
6. Regulatory compliance and ethical considerations: Compliance with regulatory requirements, such as those set by medical authorities or data protection regulations, is addressed to ensure the responsible and ethical deployment of the AI model. Patient consent, privacy, and ethical considerations are given utmost importance [59].
7. Monitoring and maintenance: Deployed AI models are continuously monitored to assess their performance, detect any anomalies, and identify opportunities for improvement [60,61]. Regular maintenance activities, including updating the model with new data, retraining, or refining its algorithms, are carried out to keep the model up-to-date and effective.
8. Collaboration and feedback: Collaboration between AI experts, healthcare professionals, and stakeholders is encouraged to gather feedback, address concerns, and optimize the AI model’s performance for better patient outcomes and clinical decision-making [62].

The deployment strategy must ensure the transparency, interpretability, and safety of AI systems in healthcare while also maintaining the expertise and judgment of healthcare professionals in the decision-making process. Continuous bias monitoring is essential as deployment conditions differ from training environments, where demographic and socioeconomic factors can reintroduce biases. We propose post-deployment fairness audits and feedback loops incorporating healthcare professionals and patients to identify and correct discrepancies between model predictions and real-world outcomes, particularly for underrepresented populations.

## 3. Discussion

AI solutions for biomedical problems are developed within scientific, commercial, and ethical frameworks, which influence their design, deployment, and outcomes [63]. These systems are shaped not only by scientific interest, but also by practical constraints, including data availability and ethical considerations such as fairness and privacy. For example, models that perpetuate biases risk harming marginalized populations and undermining trust in AI-driven healthcare. Ethical AI design mandates participatory approaches, involving diverse stakeholders—patients, clinicians, and policymakers—in every stage of development. Transparency is equally crucial, with open reporting on model limitations and potential biases.

Simply stating that biased data affect model outcomes is insufficient for understanding the full impact on medical decision support. Biases in data often mirror societal inequities, such as underrepresentation of minority groups in clinical trials, geographic disparities between urban and rural healthcare facilities, and socioeconomic barriers to accessing medical care. These variations in data collection methodologies and healthcare access create systemic biases that affect different regions and socioeconomic groups disproportionately. These biases can lead to algorithmic discrimination, where AI models perpetuate existing inequities by providing inaccurate or less effective recommendations for marginalized populations. Therefore, addressing bias requires a more holistic approach that goes beyond technical fixes in data preprocessing or performance metrics. It involves rethinking how data is collected, curated, and applied to ensure more equitable AI outcomes.

### 3.1. Intelligent Decision Support Systems: Beyond Model Deployment

While our initial recommendations focused on trustworthy AI model evaluation and deployment, we acknowledge that this approach does not fully encompass the complexity of intelligent decision support systems (IDSSs) in healthcare. An IDSS integrates multiple components beyond a deployed model, including data preprocessing pipelines, human–computer interfaces, knowledge bases, and reasoning engines, creating an adaptive, context-aware system for complex decision-making environments. A well-designed IDSS must incorporate mechanisms for integrating clinical expertise, patient data, and real-time feedback from healthcare professionals. It should facilitate collaborative decision-making by providing interpretable results, confidence scores, and clear reasoning pathways. Moreover, an IDSS should account for the dynamic nature of medical practice, where new data, evolving guidelines, and individual patient preferences.

To address these requirements, we propose expanding our design methodology to include the following components:

- Knowledge management systems: To store and update clinical guidelines, medical literature, and historical patient outcomes.
- Clinical workflow integration: To ensure that the AI’s outputs are seamlessly integrated into the medical professionals’ workflow, providing recommendations at the point of care.
- Interactive user interfaces: Allow clinicians to understand the model’s reasoning and adjust parameters or provide feedback, enhancing trust and interpretability.
- Patient-centric components: Such as decision aids that provide patients with understandable explanations of their options, fostering shared decision-making between patients and healthcare providers.

While the traditional steps for deploying an AI model are necessary, they are just one part of the broader ecosystem required for a truly intelligent medical decision support system. By refining our design methodology to include these additional elements, we can create systems that not only improve decision accuracy, but also ensure that medical AI is both transparent and adaptable to the complex realities of healthcare.

### 3.2. Expanding Bias Mitigation Beyond Model Performance

Addressing structural biases inherent in the healthcare system is essential when deploying AI systems. These systems interact with biased policies, organizational practices, and resource disparities, necessitating a multi-dimensional approach to bias mitigation:

- Auditing data sources for systemic biases (e.g., underrepresentation of certain populations).
- Tracking performance across subgroups to ensure that models perform equitably for all patients.
- Contextualizing AI outputs within the broader healthcare environment to ensure that recommendations align with ethical and clinical best practices.
- Engaging diverse stakeholders, including patients and healthcare providers from underrepresented groups, in the development and evaluation of AI systems.
- Implementing ongoing monitoring and feedback mechanisms to identify and address emergent biases in real-world settings.

Furthermore, we propose that bias mitigation should be integrated into every stage of the AI lifecycle, from problem formulation and data collection to model deployment and ongoing evaluation. This holistic approach requires collaboration between AI developers, healthcare professionals, policymakers, and patient advocates to ensure that AI systems contribute to fairer and more inclusive medical decision support.

### 3.3. Algorithm Sharing and Technology Reuse

The sharing of algorithms and trained models has gained significant traction in machine learning and deep learning, offering potential for accelerated progress and efficient resource utilization [64]. However, this practice presents ethical and intellectual property (IP) concerns that require careful consideration, particularly in the context of medical decision support systems [65].

Sharing algorithms can have profound consequences for privacy, bias, and discrimination [66]. In medical contexts, where decisions can directly impact patient outcomes, these concerns are particularly acute. An algorithm trained on biased medical data could perpetuate health disparities if shared and implemented widely [67]. Moreover, the use of shared algorithms in sensitive medical decision-making raises issues of transparency and accountability.

Algorithms, like any other software, can be protected by copyright, patents, and trade secrets, potentially leading to conflicts regarding ownership and licensing. Open-source licenses offer one effective approach, enabling algorithm sharing while protecting creators’ rights [68]. For example, the GPL license requires that any derivative works of the original code must be licensed under the same terms, ensuring that the code remains free and open. Several organizations, such as the IEEE, ACM, and Partnership on AI, have formulated guidelines for ethical AI development and deployment [69]. These guidelines cover aspects such as transparency, accountability, fairness, and privacy, providing a framework for the responsible sharing of algorithms.

In healthcare, these approaches must be tailored to address the unique challenges of medical data, including patient privacy, regulatory compliance, and the potential impact on human life. Striking the right balance between openness and protection in medical algorithm sharing is crucial for advancing healthcare innovation while safeguarding patient interests [70].

### 3.4. Recommendations

Our discussion on the model creation process has highlighted that traditional methods alone are inadequate in addressing challenges stemming from bias and noise in medical AI. Model creation, performance measurements, and benchmarking heavily rely on data availability, which can perpetuate existing biases. We propose moving beyond purely data-driven approaches to incorporate a problem-solution design methodology for fairer and more inclusive AI solutions. We formulate this as a design problem, aiming to reduce biases arising from data, algorithms, and user interaction. The innovation is that data-driven AI model creation is just the first of three steps in a more comprehensive process, requiring continuous refinement toward a practical, trustworthy, and clinically validated solution.

**Figure 2:**
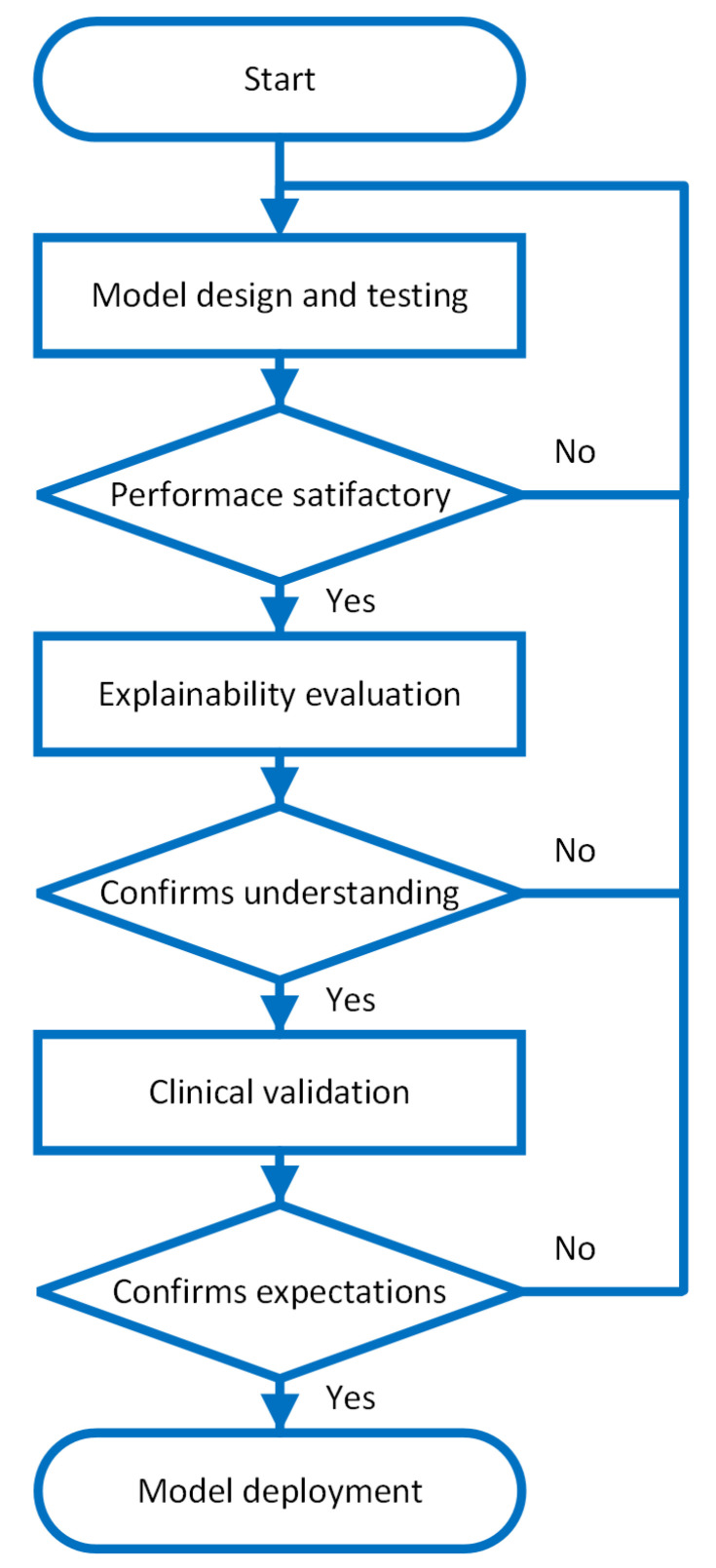
Flow diagram of the proposed AI model design methodology for medical decision support systems. The process consists of three major stages: (1) Model Design and Testing, which involves iterative data-driven development and empirical performance evaluation; (2) Trust Building, where explainability analysis is used to verify alignment between AI decisions and human mental models; and (3) Clinical Validation, which assesses the model’s effectiveness in providing medical decision support. The feedback loops indicate that failing to meet criteria at any stage requires returning to previous stages for refinement.

Figure 2 depicts our proposed design methodology. The “Model design and testing” block represents the data-driven approach outlined in the previous section. Empirical performance evaluation and benchmarking will help us to determine if the model is promising and if we can progress in the design methodology. If this is not the case, it is necessary to go back and refine the data-driven AI model. The “Trust building” step transitions into social and clinical science through explainability analysis, testing if the AI model aligns with human mental models. Only trustworthy models progress to clinical validation, where we establish if the model provides efficient medical decision support. Passing these tests indicates sufficient uncertainty reduction for practical deployment.

After outlining the general steps to design fairer and more inclusive medical decision support systems, we now focus on specific design patterns and best practices that should be followed during the design process. Good documentation is essential for successful system design because it enables reproducibility and helps to assess noise and biases, which builds trust in the system. The creation of the AI model should be described in detail, including the technical implementation and performance testing. During the deployment phase, it is crucial to validate the performance of the model in a practical setting. The processes underlying the block descriptions should be clearly explained, specifying the methods employed. Failure to provide detailed descriptions of the methods utilized constitutes a distinct limitation of any study on medical decision support, and it should be explicitly acknowledged.

We recommend incorporating the following features into the training and testing processes:

- Multicenter data: Utilizing data from multiple centers can enhance the model’s generalization and make it more robust to variations in data collection protocols and equipment. Applying this technique can reduce measurement bias [71].
- Standardization of preprocessing reporting: Documenting the preprocessing steps employed in preparing the data for model training is important. Standardization of preprocessing reporting ensures the reproducibility of experiments. This can help to explain and subsequently address data-related bias.
- Annotated data from multiple operators: Multiple experts or operators in the process of annotating or labeling medical data for training machine learning models or decision support systems. This approach is utilized to mitigate bias, increase accuracy, and ensure diverse perspectives in the annotation process [72].
- Performance reporting standards: Using standard performance metrics relevant to the specific task is crucial for assessing the level of noise in a given dataset and reporting the performance accurately [73].
- Reproducibility: Sharing the source code alongside the dataset, if feasible, can enhance AI methods [74]. By openly providing the source code, researchers and practitioners can replicate and validate the results, ensuring transparency and promoting scientific rigor. Furthermore, sharing the dataset enables other researchers to evaluate and compare different algorithms on the same data, facilitating a comprehensive understanding of the methods’ performance and potential biases.
- Explainability: To foster trust and confidence in the model, it is essential to provide explanations for its predictions. Methods such as LIME, SHAP, and Grad-CAM [75] can be employed for explainability [76]. Explainable AI models aid in identifying biases and understanding the decision-making process, enabling stakeholders to effectively address potential biases.
- Uncertainty quantification: Estimating uncertainty in the model’s predictions is crucial, especially in medical applications where incorrect predictions can have serious consequences. Recent papers addressing uncertainty quantification [77,78] offer valuable insights into this domain.
- Continuous monitoring and evaluation: Regular monitoring of the system’s performance and evaluation for potential biases is crucial [79]. Ongoing assessment can help identify and rectify any bias that emerges over time, enabling the system to adapt and improve its fairness and accuracy.

By incorporating these features, the model development process can be enhanced, resulting in more reliable and accountable AI systems in the medical domain. Figure 2 displays the development diagram of an AI model incorporating all the mitigation measures proposed in this work.

### 3.5. Limitations and Future Work

Some paragraphs in our position paper may appear vague due to the necessary abstraction required to address broad, systemic issues. Introducing specific technical or methodological details would lead to incompleteness and inconsistency. For instance, discussing CT-based lesion detection would necessitate focusing on a particular medical field—such as oncology, neurology, or pulmonology—each requiring detailed exploration which exceeds the scope of this manuscript. The abstraction enables us to highlight important challenges of bias and noise in biomedical AI. Historically, progress in medicine relied on standardization and education, both subject to systemic noise and bias. AI models, however, allow us to simulate “what-if” scenarios, providing some insights that might help to quantify and mitigate these challenges in contexts like medical decision support. By maintaining this level of abstraction, we aim to provide a clearer reflection on the limitations and opportunities of AI in fostering fair and inclusive biomedical solutions.

In future we anticipate that understanding data sources in terms of noise and bias will become increasingly crucial. Different measurement environments introduce varying levels of noise and bias. For example, clinical measurements of the electrical activity of the human heart using a 12-lead ECG exhibit less noise compared to a 1-lead pickup system commonly utilized for ECG measurements in home environments. However, clinical measurements are often shorter, leading to the introduction of selection bias. Therefore, future studies should prioritize quantifying the disparities between measurement environments with standardized measures for bias and noise.

The development of new AI algorithms is an ongoing process, encompassing both domain-specific and general-purpose approaches. Moving forward, it is crucial to continue exploring a wide range of AI algorithms rather than confining ourselves to dedicated solutions solely for medical decision support. For instance, it may be feasible in the future to provide medical decision support in home environments by executing lightweight AI models on edge devices. This approach offers potential benefits, such as reducing selection bias through prolonged observation durations. However, if the edge device operates on battery power, the runtime will be influenced by the computational complexity of the AI model. Opting for a lightweight model would result in a longer runtime, thereby enhancing the usability of such solutions. An illustrative example is the development of an AI model for predicting cardiovascular risk. Incorporating federated learning enabled the use of data from multiple regions while preserving privacy. Stratified sampling helped address geographic disparities, ensuring that the model performed equitably across diverse populations.

From a medical perspective, disease-specific decision support systems within diagnostic pathways require tailored solutions with significant implications for noise and bias. Unfortunately, research exploring disease-specific bias and noise remains limited. Different performance measures can impact the trustworthiness and deployability of AI models in diverse ways, necessitating detailed examination of these aspects.

Considering individual diseases as isolated occurrences is a simplistic approximation of medical scenarios. Many clinical scenarios involve co-morbidities, which makes disease diagnosis more complex. Therefore, future studies should incorporate co-morbid data in the testing and evaluation of AI models, such that the training and testing regime reflects complex clinical scenarios. This enables more comprehensive evaluation of model capabilities and potential biases while acknowledging that multiple diseases often coexist. This framework leads to a crucial extension for medical decision support systems: the ability to identify multiple conditions simultaneously. For example, it might be feasible to identify cardiovascular diseases and sleep disorders by observing the electrical activity of the human heart. The task of multi-disease classification could be facilitated by using multi-modal data [80]. Such data can be captured with a wireless body area network that integrates a wide range of physiological sensors.

## 4. Conclusions

The development of fair and inclusive AI for medical decision support systems is a complex challenge that requires a multifaceted approach. Throughout this paper, we have explored the various sources of noise and bias in medical data and AI models, and proposed strategies to mitigate these issues.

We conclude that the validity and usefulness of medical data can be significantly enhanced by implementing a comprehensive set of measures such as study design, appropriate statistical analysis, transparent reporting, and promoting diverse representation in research. Moreover, our proposed design methodology, which extends beyond traditional data-driven approaches, offers a framework for creating more robust and reliable AI models for medical decision support.

## Data Availability

No new data were created or analyzed in this study.
